# From Function to Phenotype: Impaired DNA Binding and Clustering Correlates with Clinical Severity in Males with Missense Mutations in *MECP2*

**DOI:** 10.1038/srep38590

**Published:** 2016-12-08

**Authors:** Taimoor I. Sheikh, Juan Ausió, Hannah Faghfoury, Josh Silver, Jane B. Lane, James H. Eubanks, Patrick MacLeod, Alan K. Percy, John B. Vincent

**Affiliations:** 1 Molecular Neuropsychiatry & Development (MiND) Lab, Campbell Family Mental Health Research Institute, Centre for Addiction and Mental Health, Toronto, ON, Canada; 2 Institute of Medical Science, University of Toronto, Toronto, ON, Canada; 3 Department of Biochemistry and Microbiology, University of Victoria, BC, Canada; 4 The Fred A. Litwin Family Centre in Genetic Medicine, University Health Network and Mount Sinai Hospital, Toronto, ON, Canada; 5 Civitan International Research Center, University of Alabama at Birmingham, AL, USA; 6 Division of Genetics and Development, Toronto Western Research Institute, University Health Network, Toronto, ON, Canada; 7 Department of Physiology, University of Toronto, Toronto, ON, Canada; 8 Department of Surgery (Neurosurgery), University of Toronto, Toronto, ON, Canada; 9 Department of Medical Genetics, University of British Columbia, Vancouver, BC, Canada; 10 Department of Psychiatry, University of Toronto, Toronto, ON, Canada

## Abstract

Mutations in the *MECP2* gene cause Rett syndrome (RTT). MeCP2 binds to chromocentric DNA through its methyl CpG-binding domain (MBD) to regulate gene expression. In heterozygous females the variable phenotypic severity is modulated by non-random X-inactivation, thus making genotype-phenotype comparisons unreliable. However, genotype-phenotype correlations in males with hemizygous*MECP2* mutations can provide more accurate insights in to the true biological effect of specific mutations. Here, we compared chromatin organization and binding dynamics for twelve MeCP2 missense mutations (including two novel and the five most common MBD missense RTT mutations) and identifiedacorrelation with phenotype in hemizygous males. We observed impaired interaction of MeCP2-DNA for mutations around the MBD-DNA binding interface, and defective chromatin clustering for distal MBD mutations. Furthermore, binding and mobility dynamics show a gradient of impairment depending on the amino acid properties and tertiary structure within the MBD. Interestingly, a wide range of phenotypic/clinical severity, ranging from neonatal encephalopathy to mild psychiatric abnormalities were observed and all are consistent with our functional/molecular results. Overall, clinical severity showed a direct correlation with the functional impairment of MeCP2. These mechanistic and phenotypic correlations of MeCP2 mutations will enable improved and individualized diagnostics, and may lead to personalized therapeutic interventions.

Rett syndrome (RTT; MIM#312750) is an X-linked neurological disorder present in ~1:10,000–15,000 girls^1^. RTT children often appear to develop normally in early infancy; however, changes in patterns of mental and social development usually begin to appear between 6 and 18 months of age. Slower head growth and loss of muscle tone are common early symptoms, along with more general problems with gross motor development and coordination. Other clinical features and symptoms may include repetitive hand movements, scoliosis, constipation, excessive saliva, intellectual disability, stiff-legged gait, periodic breathing, seizures, and typically little or no verbal skills. Although RTT is known to be caused by dysfunction of the methyl-CpG-binding protein-2 gene, *MECP2*, there is a broad range in severity and co-morbidities. In affected heterozygous females, phenotypic severity may vary with patterns of non-random X chromosome inactivation across the brain and other relevant tissues^2^. Detailed clinical criteria used for RTT diagnostics are described elsewhere^3^.

*MECP2*, on Xq28, was identified in 1999 as the gene responsible for RTT^4^. MeCP2 was initially identified due to the selective binding to DNA sequences with methylated CpG dinucleotides^5^,^6^, however, MeCP2 has also shown moderate binding affinity to un-methylated DNA^7^. MeCP2 competes with histone H1 and binds linker DNA^8^,^9^, which suggests that MeCP2 and histone H1 may share similar functions with respect to chromatin organization. MeCP2 is a 60–80% unstructured, intrinsically disordered protein that shows coil-like hydrodynamic properties in solution^8^,^10^. The hydrophobic patch within the MBD forms hydrophobic interactions with methylated CpG. The X-ray crystallographic structure of the MBD bound with DNA has highlighted unique structural roles for various residues, which may be important in the DNA-binding dynamics of MeCP2^11^ (Fig. 1A).

Mutations in the MeCP2 gene are commonly linked to RTT, but MeCP2 has also been associated with a number of other developmental disorders, including in males^12^. This suggests that it may play a central role in the post-natal development of the human brain^13^. There are ~160 different single nucleotide variations (SNVs) that have been reported in ~1593 patients, out of which ~943 (~59.01%) have 60 different SNVs in the MBD^14^. A more comprehensive understanding of the functional effects of MeCP2/MBD missense mutations and genotype/phenotype will assist the search for therapeutic compounds targeting these deficits, as well as enable a clearer picture of the downstream molecular and/or cellular disruptions in common between RTT patients. T158, R133, R106 and P152 are common Rett related mutation hotspots within the MBD of MeCP2. According to Rettbase, among MBD missense mutations, Thr158Met is the most common (8.79% of reported cases, also the most common mutation overall), followed by R133C (4.24%), R106W (2.76%), P152R (1.41%) and A140V (0.66%)^14^. T158M has been reported in a few hemizygous male patients^15^,^16^,^17^. P152R has been reported as a pathogenic variant only in females, showing classical, typical, and atypical Rett symptoms. In a single study, P152A was reported as a hypomorphic mutation in a male patient^18^. R106, R111, N126, R133, A140, P152, F157, T158 and R167 are conserved across all vertebrates (Fig. 1B), and substitution at these structurally important residues may affect the ability of MeCP2 to bind properly to its interacting targets, which may affect gene expression regulation and/or chromatin organization as well as its mobility in the cellular environment. Here, using cells transfected with several mutant MeCP2-GFP constructs in order to model the different mutations as a more practical approach than using patient-derived cells, we studied: 1) the effect of rare and common MBD mutations on chromatin organization and on DNA-binding kinetics; and 2) the correlation between clinical severity and the cellular effect for males with the MBD missense mutations N126I, R133C, A140V, P152A, P152H, F157I, T158M and R167W.

## Results

### Study participants’ history and clinical characteristics

Detailed patient ascertainment and clinical descriptions are in the supplementary material. A comparison of clinical conditions of males with the hemizygous MeCP2 mutations N126I, R133C, A140V, P152A, P152H, F157I, T158M, and R167W are highlighted in Table 1.

### MeCP2 localization at chromocenters

Figure 2A represents confocal stacks of all recombinant MeCP2 mutant and control proteins (GFP), DNA (counterstained with DAPI), and merged images representing the patterns of co-localization of both fluorophores. Although each mutant represents its unique binding and clustering pattern, we have divided the patterns into two scenarios, 1) mutants such as R111G, R133L and R106W showing complete mislocalization, likely due to impeded binding of mutant MeCP2 with DNA; 2) mutants such as P152R, F157I and N126I showing higher number and decreased size of chromocenters, likely due to impaired chromatin compaction (see below).

Merged images of chromatin DNA (DAPI) and MeCP2 fusion protein (GFP) for R111G, R106W, R133L and T158M show a small number of distinctively identifiable and clustered chromocenters (Fig. 2A). In the quantitative co-localization analysis of transfected MeCP2 and chromatin, median Pearson correlation coefficient (PCC) values (*r*_p_) for the R111G, R106W, R133L and T158M constructs were calculated as *r*_p_ = 0.26, 0.40, 0.46 and 0.57 respectively (p ≤ 0.0005). In contrast, constructs for P152R, F157I, P152H, P152A, N126I, R133C, P152A and R167W had PCC values of *r*_p_ = 0.68, 0.69, 0.71, 0.72, 0.72, 0.82 and 0.81 respectively (Fig. 2B,C). PCC values of *r*_p_ ≤ 0.60 were not considered as mislocalized (Fig. 2C). Overall, we observed three different patterns among mutants, 1) impaired clustering; 2) abolished clustering; and 3) abolished binding (Fig. 2D).

### MeCP2 binding at chromocenters

From the co-localization analysis of chromatin and mutant MeCP2, we found abolished co-localization for mutants such as R111G, R106W, T158M and R133L as mutant protein unable to localize at the chromocenter (Fig. 2A–D). Any further quantitative analysis of these mutants was not possible. In the quantitative analysis, comparisons of number and size of distinct identifiable chromocenters are shown in graphical form (Fig. 3). *P*-values (two tailed Mann Whitney non- parametric U test) are indicated where statistically significant (<0.05) for each MeCP2 mutation in comparison with WT MeCP2. In the initial quantitative analysis we compared transfected and untransfected cells using the DAPI channel to quantify chromocenters, and the GFP channel to quantify recombinant MeCP2_E1. Chromocenters of transfected cells over-expressing MeCP2_E1 showed significant clustering of chromocenters in comparison with untransfected cells (Fig. 3A). Quantitative number and size analysis reveal more but significantly smaller sized chromocenters in the untransfected cells (Fig. 3B).

We also compared number and size of chromocenters of wild type MeCP2_E1 and all mutant MeCP2 constructs included in this study. Due to extremely low quantity of distinctly identifiable chromocenters, R111G, R106W, R133L and T158M were not considered in the chromocenter number and size analysis. This lack of identifiable chromocenters suggests either loose or no binding, in addition to mislocalization, leading to a complete loss of chromatin clustering (Fig. 2). The chromocenter number and size for R133C was also slightly lower than WT, but non-significant (two tailed Mann Whitney non parametric U test *p* value ≥0.05; Fig. 3C,D).

Cells expressing N126I, A140V, P152R, P152H, F157I and R167W MeCP2 showed a significant increase in the number of chromocenters (*p* ≤ 0.005). For P152A, on the other hand, the average chromocenter numbers were not significantly different from WT and other controls (Fig. 3C). Similarly, for the chromocenter size analysis, N126I, A140V, P152R, P152H, F157I and R167W showed a significant decrease in chromocenter size (*p* ≤ 0.005), whereas for P152A chromocenter size was not significantly different from WT and other controls (Fig. 3D).

### Real-time recovery and binding dynamics of Rett mutants

To further study the effect on protein mobility and binding dynamics, we selected several mutants that were typical for the distal category such as 1) P152A, 2) P152H and 3) P152R and two mutants typical for the proximal category such as T158M and R111G. Chromocenter fluorescence recovery after photobleaching (FRAP) was used to assess the binding dynamics of MeCP2 with the following missense mutations: P152A, P152H, P152R, T158M and R111G, in comparison to WT (Supplementary Videos). Figure 4A,B shows pre-bleach, bleached and post-bleached recovery images. R111G, T158M and P152R constructs showed a significantly more rapid rate of recovery (two tailed Mann Whitney non parametric U test; *p* ≤ 0.05). Almost complete recovery was observed before the 60^th^ frame, whereas for WT the recovery rate was slow and incomplete by the 160^th^ frame. Values of recovery rates forP152A and P152H were not significantly different to WT. Slower recovery suggests tighter binding with heterochromatin, whereas faster recovery suggests looser binding, e.g. for T158M, R111G and P152R. However, concentration of available MeCP2 protein within the nucleus could also be influencing these rates. A comparative analysis of the averaged values of quantitative parameters t-half and mobile fraction is shown (Fig. 4D,E).

To evaluate statistically how well the model fits the data, *R*^2^ values of each FRAP curve were calculated. Averaged (n = 5, independent FRAP experiments) value of the square of the correlation (*R*^*2*^) between the response values and the predicted response values (goodness-of-curve-fit) were *R*^2^ = 0.992 for WT, 0.99 for P152A, 0.976 for P152H, 0.8 for P152R, 0.948 for T158M, 0.674 for R111G and 0.982 for PP390-91del constructs. Averaged t-half recovery was ~64.16+/−23.11 sec, ~52.61+/−2.27 sec, ~42.35+/− 23.79 sec, ~12.44+/− 3.86 sec, ~12.74+/−16.76 sec, 15.85+/−8.1 sec and 50.86+/−22.69 for MeCP2 WT, P152A, P152H, P152R, T158M, R111G and PP390-91del constructs, respectively (Fig. 4D). The t-half for P152R, T158M and R111G was much lower than that for WT. Similarly, the numeric value of the mobile fraction (normalized to one) was ~0.59+/−0.07, ~0.61+/−0.15, ~0.56+/−0.16, ~0.69+/−0.13, ~0.79+/−0.12, 0.92+/−0.09 and 0.69+/−0.23 for MeCP2 WT, P152A, P152H, P152R, T158M, R111G and PP390-91del constructs, respectively (Fig. 4E).

Table 1 shows phenotypes in several very rare cases of hemizygous male patients with MeCP2 mutations. Clinical presentation such as condition at birth, age of reported first abnormality, history of development, head circumference, muscular abnormalities, motor skills, epileptic seizures, language and social skills, age and cause of death (if deceased) were compared and reported here. Interestingly, a wide range of phenotypic/clinical severity, ranging from neonatal encephalopathy to psychiatric disorders were observed. To determine the severity of clinical manifestations of the study participants, we used the clinical severity score(CSS), developed previously^19^. CSS were calculated and plotted in Fig. 5A. Where the same mutation was reported for multiple participants, the average CSS across these participants was plotted. Parameters such as chromocenter number (CN) and size (CS) were used where possible (i.e. where the mutation did not completely abolish chromocenter clustering, as seen for T158M, or binding as seen for R111G and R133L) to calculate Functional Severity Score (FSS) using the equation 1 below and were correlated with CSS (Fig. 5B,C).


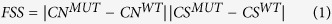


## Discussion

We used C2C12 cells, which express relatively low levels of endogenous MeCP2 and have prominent heterochromatin/chromocenters suitable for qualitative and quantitative analysis of DNA binding proteins, with the aim of studying the effect of common and rare Rett MBD missense substitutions on the binding and clustering of the chromatin. The alternative approach involving studying the binding in patient-derived cells for each mutation is possible, but impractical. Using transfection of MeCP2_GFP fusion constructs presented us with the opportunity to expand the study to include a much larger number of mutations than in similar previous studies. It should be noted that the MeCP2-GFP fusion itself is unlikely to have different properties compared to native MeCP2, since transgenic mice expressing MeCP2-GFP are viable and almost undistinguishable from the wild type mice^20^. In particular, we were interested in studying how the positional difference of each mutant in the tertiary structure of the MBD of MeCP2 affects its binding to the heterochromatin and mobility in the nuclear environment.

In the quantitative co-localization, the high degree of mislocalization of R111G, R106W and R133L MeCP2 suggest that MeCP2 is not binding at the chromocenters (Fig. 2B,C). Complete loss of the specific binding of MeCP2 to methylated DNA has already been reported for the R111G substitution^21^. R106W is present on the β-sheet site directing towards the DNA binding residue R111, which may disrupt the conformation of the MeCP2 DNA-binding pocket. For T158M, relatively higher PCC, indicates either a partial loss of binding or a complete loss of chromatin clustering (Fig. 2A,C). Overall, we can characterize these changes in the chromatin binding and clustering into three different patterns among mutants, 1) impaired clustering; 2) abolished clustering and 3) abolished binding (Fig. 2B).

**Figure 1:**
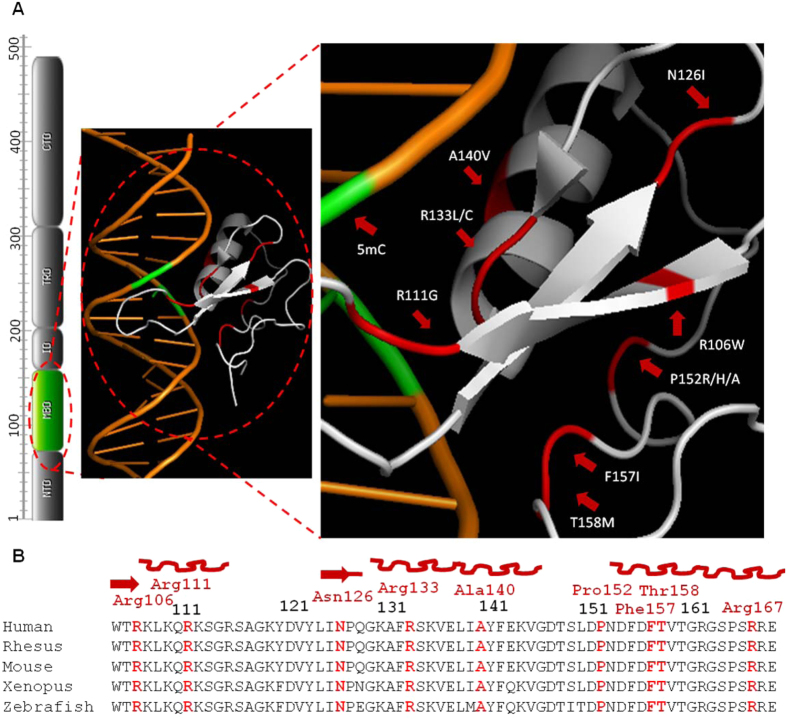
Structural analysis of human MeCP2 MBD bound at brain-derived neurotrophic factor (BDNF) promoter. (**A**) MeCP2 domain structure with approximate amino acid number in each domain^39^ and the only highlighted domain (green) is MBD. (**B**) Human MECP2-MBD crystallographic structure^11^ illustrated using PyMol Molecular Graphic System (PDB accession code: 3C2I) showing MBD bound at methylated double stranded DNA sequence of BDNF promoter. Location of amino acids substituted such as R106W, R111G, N126I, R133C/L, A140V P152A/H/R, F157I, T158M are indicated with arrows (highlighted as Red). (**C**) Multiple sequence alignment (MSA)^40^ of the full sequence of MBD of MeCP2 of five vertebrates (NCBI GeneBank accession numbers: *Homo sapiens* gi|1708973|sp|P51608.1, *Macaca fascicularis* gi|50401118|sp|Q95LG8.1, *Mus musculus* gi|12585281|sp|Q9Z2D6.1, *Xenopus laevis* gi|4105999|gb|AAD02651.1, and *Danio rerio* gi|62204887|gb|AAH93116.1) to show conservation at the MeCP2 missense mutation residues (red).

Cells expressing N126I, A140V, P152R, P152H, F157I and R167W MeCP2 showed a significant increase in the number of chromocenters. Agarwal *et al*.^22^ previously reported severely affected heterochromatin clustering in proline substitutions P101R, P101H and P152R. Similarly, Adegbola *et al*.^18^ reported disrupted MeCP2-heterochromatin binding for P152A, P152R and T158M. Here, we confirm these observations and further report two novel substitutions, P152H and N126I, which also affect the clustering of heterochromatin. In the case of P152A, chromocenters were normal in number and size (Fig. 3C,D), and with relatively normal clustering of chromocenters, showing that some substitutions at P152 may have relatively minor consequences for DNA-clustering or compaction. Analysis of chromocenter numbers across all the mutants and WT showed a roughly two-fold range of variability (Fig. 3C). Interestingly, all the mutants that show a clustering defect (i.e. N126I, A140V, P152R, P152H and R167W) are located within the distal part of the MBD (Fig. 1A). This indicates the importance of the role of MBD, not only in binding to DNA, but also in the clustering of chromatin. It is not yet known whether MeCP2 performs this chromatin clustering and organizational function alone, or whether it is also dependent on interactions with its protein partners. However, it has been suggested previously that the MBD mutation A140V, which is present in the same distal pocket as P152R (Supplementary Fig. S1), loses the ability to interact with ATP-dependent helicase, ATRX, which is an important chromatin remodeling protein^23^. These findings underpin the important architectural role of MeCP2 in the organization of chromatin.

There were no significant differences in the chromocenter size and number for the A2V (N-terminal) and PP390-91del (C-terminal) mutants compared with wild type MeCP2, suggesting that the patho-etiology in Rett patients with these mutants is independent of the MBD (Fig. 3C,D). For A2V, *in silico* analysis using Termi*N*ator (http://www.isv.cnrs-gif.fr/terminator3/) predicts decreased N-terminal methionine excision and N-acetylation and decreased stability/longevity of the protein^24^, which most likely plays an important role in MeCP2 homeostasis. For PP390-91del there may be an effect on the binding of MeCP2 to other proteins due to deletion in the proline motif at the C-terminal domain (CTD)^25^.

MeCP2 mutants such as R111G, T158M and P152R showed significantly higher recovery rates than wild type MeCP2, suggesting altered diffusion rate and mobility dynamics of the mutant protein. Previously, Kumar *et al*.^26^ described the influence of different MeCP2 domain and RTT mutations on the DNA binding and clustering of chromatin using the same photobleaching approach. Here we further extended the study by using a site specific approach to describe how the binding and mobility dynamics of MeCP2 vary due to chemical and physical properties of the substituting amino acid, and how these differences relate to the clinical phenotypes in hemizygous males. R111G, T158M and P152R showed extremely fast recovery times (p ≤ 0.05). Also, mobile fraction of R111G and T158M is significantly higher than wild type, whereas for P152R no significant amount of MeCP2 protein was mobile. This confirms that P152R does not affect the binding, instead it only affects MeCP2 mobility within the cellular environment. In addition, it is clear that different substitutions at P152 have different effects at the molecular level. Even though the recovery rates (t half) for P152H and P152A are not significantly different, they were somewhat faster than wild type, which may go some way to explain the milder phenotypes associated with these mutations. Interestingly, for the first time a clear correlation can be seen with average t-half recovery time in comparison to the clinical severity score (CSS) for the respective patients, with increasing severity: WT < P152A < P152H < P152R < T158M < R111G (Table 1; Figs 4D,E and 5A) and overall the clinical severity correlates highly with our functional/molecular tests, i.e. from mildest to severest effect: MeCP2 WT > P152A > A140V > R167W > P152H > median CSS for RTT girls > N126I > R133C > F157I > T158M (Fig. 5A,B).

To help explain the effects of the substitutions at various specific positions in the MBD tertiary structure (Fig. 1A and Supplementary Fig. S1), we compared the physical and chemical properties of the WT versus substituted amino acids (Supplementary Table S1) (www.ncbi.nlm.nih.gov/Class/Structure/aa/aa_explorer.cgi). For instance, the difference in the DNA binding at R111 and at R133 can be explained by the different chemical properties of the substituting amino acids. Arginine is one of the most chemically complex amino acids due to its enhanced ability to mediate molecular interactions. Guanidine is the functional group on the side chain of arginine, which makes arginine the most basic of the amino acids with a virtually permanent positive charge, which is crucial for binding with the negatively charged DNA backbone, and has a higher hydrogen bonding forming potential compared to other basic amino acid residues^27^. For R111G, R133L and R133C, both glycine and leucine have non-polar hydrophobic side chains, but cysteine, on the other hand, is slightly polar and hydrophilic. Thus, the effect on MeCP2-DNA interaction is greater when the substituting amino acid is glycine and leucine, but milder when substituted with cysteine (Fig. 6A and Supplementary Table S1). This may also explain the milder phenotype of the R133C male patient in comparison with other mutants directly effecting the MeCP2-DNA binding site (Table 1), and an overall less severe phenotype reported for typical and atypical Rett girls with the R133C MeCP2 mutation^28^,^29^. In addition to different chemical properties of the substitution, the effect may also be structural. For example, R106W is present on the β-sheet, directing towards the DNA binding residue R111. The substitution of arginine 106 with tryptophan may affect the MBD folding, as the non-polar hydrophobic properties of tryptophan alters both structure and charge, and thus adversely affect MeCP2-DNA binding (Figs 1A and 6B and Supplementary Table S1).

**Figure 2:**
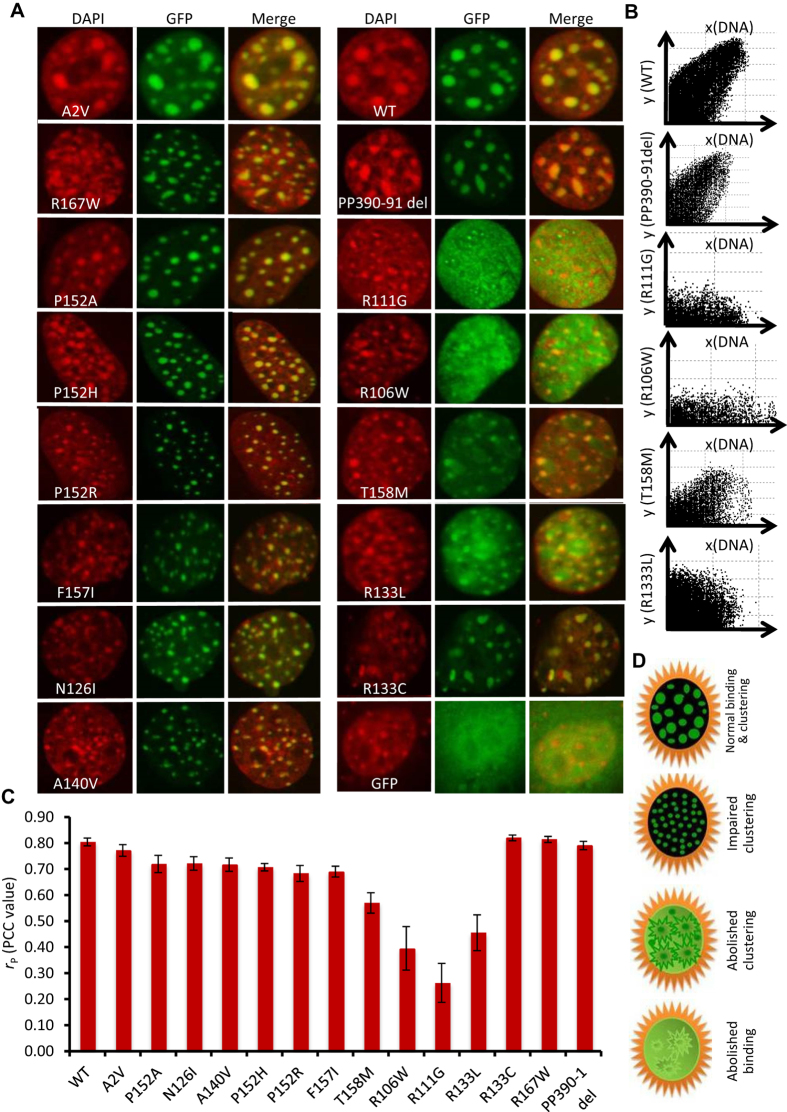
Comparison of the binding and clustering pattern of wild type and mutant MeCP2 expressing in C2C12 cells. (**A**) Confocal image stacks representing Left to right), column 1 DAPI (red), column 2 GFP (green), column 3 merge (red + green), column 4 DAPI (red), column 5 GFP (green) and column 6 merge (red + green). Left Row 1 A2V, Right Row 1 wild type, Left Row 2 R167W, Right Row 2 PP390-91del, Left Row 3 P152A, Right Row 3 R111G, Left Row 4 P152H, Right Row 4 R106W, and Left Row 5 P152R. Right Row 5 T158M, Left Row 6 F157I, Right Row 6 R133L, Left Row 7 N126I, Right Row 7 R133C, Left Row 8 A140V, and Right Row 8 GFP only. Note that all images shown here are representative of the authors’ observations; averaged data from all images are presented in the quantitative analysis, illustrated in Fig. 3 and discussed in the main text. (**B**) Scatter plot of red and green pixel intensities showing co-localization of DNA (DAPI) and recombinant MeCP2 protein (GFP). (**C**)Pearson’s correlation coefficient (PCC) *r*_*p*_values of chromocenters (DAPI) and recombinant protein (GFP) were plotted to represent quantitative co-localization of DNA and recombinant MeCP2 (n = 10, 0.6 or higher = positive co-localization; <0.6 weak positive co-localization; ≤0.5 No or negligible co-localization); +/− SEM is shown.(**D**) Cartoons shown (Right → bottom) different binding and clustering patterns with normal binding and clustering (Top, wild type), impaired clustering (Middle 1, P152R/ N126I), completely abolished clustering (Middle 2, F157I/T158M) and abolished binding (Bottom Right, R111G/R133L).

**Figure 3:**
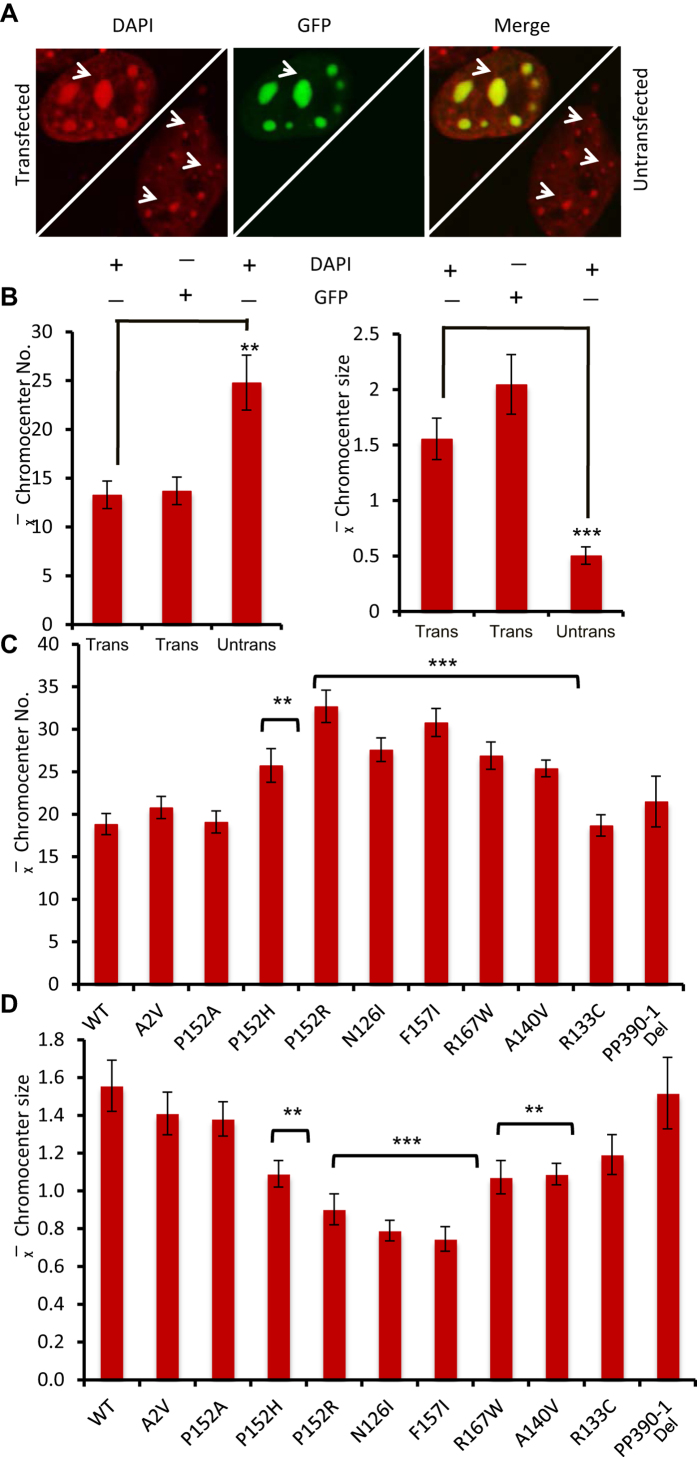
Quantitative analysis of chromocenter clustering in the transiently expressed mouse myoblast cells with mutant and wild type MeCP2 protein. (**A**) Comparison of chromocenter clustering in the transiently expressing wild type MeCP2 C2C12 cell (left corner) with neighbouring untransfected cell (right corner). (**B**) Quantitative analysis of chromocenter number and size using DAPI and GFP channels (*n* = 10); (**C**,**D**) Quantitative analysis of average chromocenter number from C2C12 cells transfected with wild type and each mutant recombinant MeCP2 protein (*n* = 20). Two tailed Mann Whitney non parametric U test. +/− S.E. bars are shown. ***P ≤ 0.005 and **P ≤ 0.05 and +/− SEM is shown.

T158M is present on a regionof the MBD potentially involved in both DNA binding and clustering of chromatin(Fig. 1B). The side chain of threonine is polar and hydrophilic and contains a hydroxyl group, whereas the methionine has a non-polar and hydrophobic side chain, and includes a S-methyl thioether side chain(Fig. 6E and Supplementary Table S1). Despite T158M MeCP2 previously reported to show close to normal electrophoretic band-shift with methylated DNA^30^, the faster recovery time and high mobile fraction observed here and previously^26^ suggests reduced binding, and in addition we show here reduced co-localization with chromocenters (Fig. 2B). The substitution of a chemically opposite amino acid most likely affects a MBD site that is important for chromatin clustering as well as DNA-binding (Fig. 6A,B and Supplementary Table S1). Interestingly, at the adjacent residue, F157I has a severe effect on chromocenter clustering (Fig. 2B). This may emphasize the effect of an aromatic side chain (phenylalanine) versus an aliphatic side chain (isoleucine) at this residue in the MBD^31^. Furthermore, this substitution may also affect a possible pi-pi interaction of phenylalanine-phenylalanine (Phe-Phe stack of aromatic rings) with the nearby residue F155 (Fig. 6C and Supplementary Table S1)^36^. R167W which is a little further down from T158 and located in the intervening domain (ID) also appears to show a significant clustering defect, but the clinical phenotype is milder than T158M and F157I.

**Figure 4:**
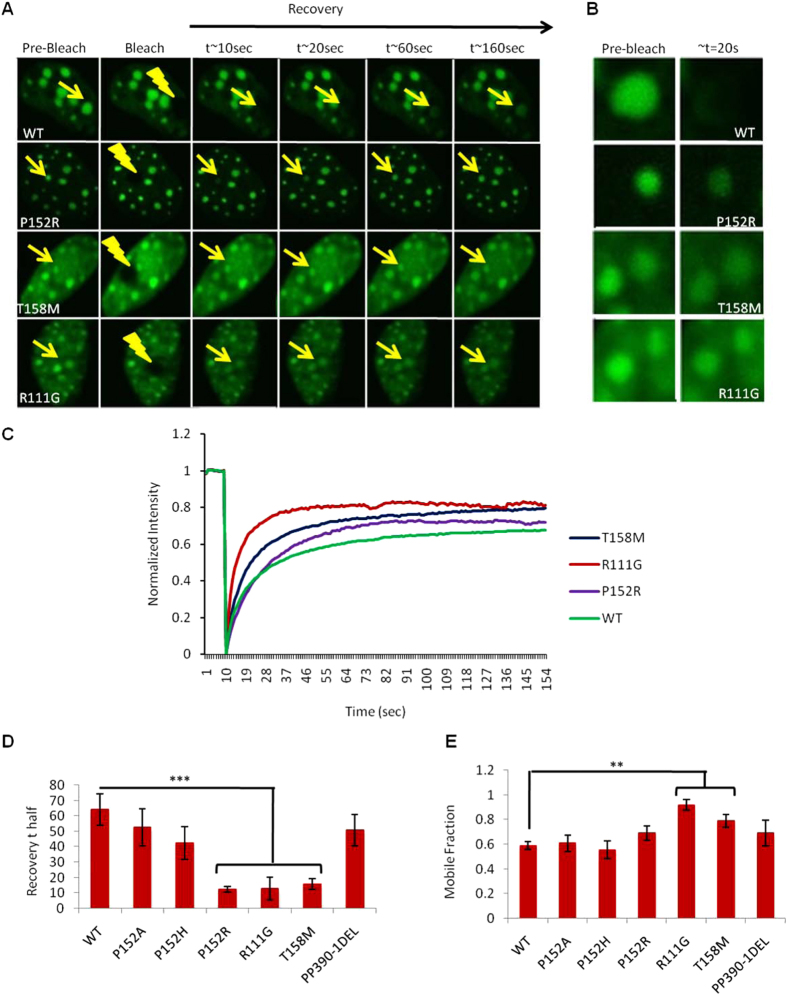
Chromocenter FRAP experiments to mobility and binding dynamics of wild type and mutant MeCP2. (**A**) Real-timepost-bleach recoveryof GFP tagged recombinant MeCP2 protein were captured after bleaching chromocenter at ~200 μsec. Yellow lightening indicate bleach spots. Rows (Top → bottom): Representation of FRAP pre-bleach, bleach and post bleach real time recovery of MeCP2 wild type, P152R, T158M and R111G, respectively. Columns (Left → Right): column 1 is representing preceding pre-bleach images; column 2 representing 1^st^ images; column 3 representing 11^th^ image; column 4 representing 21^st^ images, column 5 representing 61^th^ image and column 6 representing 161^th^ image following bleach. where, 1 frame = 108.784 m.sec. (**B**) Chromocenter recovery at ~t = 20 indicating different levels of recovery among wild type and mutant MeCP2; (**C**) FRAP recovery curves normalized to 1, showing chromocenter recovery in 151frames. (**D**,**E**) Comparitive illustration of half maximal recovery time and mobile fraction of wild type, P152A, P152H, P152R, T158M, R111G and PP390-91del, respectively. n = 5, ***p ≤ 0.05 two tailed Mann Whitney non parametric U test. +/− S.E. bars are shown.

**Figure 5:**
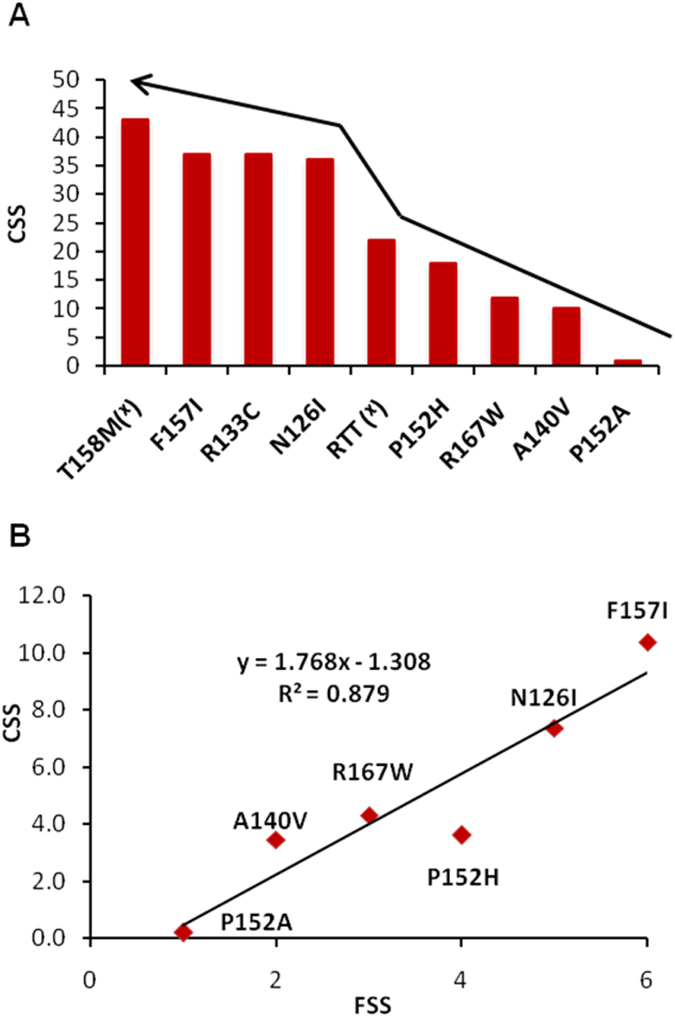
(**A**) Clinical severity scores (CSS) of F157I, N126I, P152H, P152A, ǂ median CSS value of Rett girls and T158M cases (Case I, II, III, IV in Table 1). (**B**) Scatter plot representing correlation of functional Severity Score (FSS) with clinical severity score (CSS) of MeCP2 among male cases with missense mutations, as in Fig. 6A.

**Figure 6:**
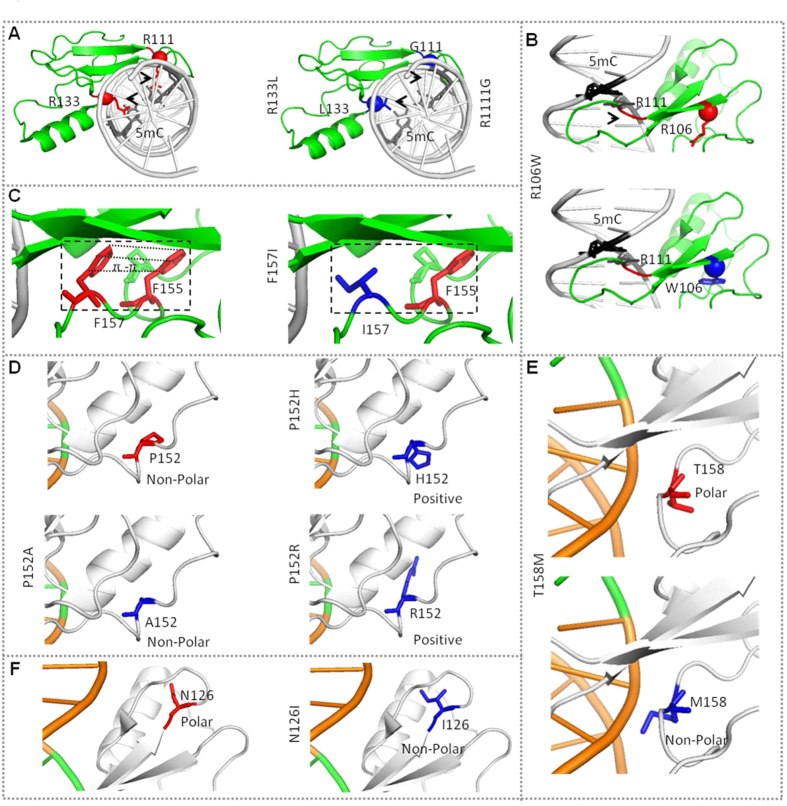
*In Silico* mutagenesis analysis of MeCP2 MBD model bound at BDNF promoter^11^ using PyMol Molecular Graphic System (PDB accession code: 3C2I), showing structural and biochemical propertiesof the wild type and substituted residue (**A**) Disruption of arginine-DNA binding site at R111G and R133L (**B**) R106W and its potential effect on R111-DNA binding site; (**C**) F157I and its potential effect on the F155-F157 interaction due to absence of the aromatic ring of F157; (**D–F**) illustration of the effectson the structural and biochemical properties of the substitutions, including (**D)** P152A, P152H and P152R; (**E**) T158M and (**F**) N126I.

Among other mutants in the same pocket of the MBD such as P152R, P152H and P152A, it should be noted that the side chain of arginine and histidine both contain a full positive charge, whereas both proline and alanine are small, non-polar and hydrophobic (Fig. 6D and Supplementary Table S1). Furthermore, proline has an exceptional conformational rigidity compared to other amino acids which may help MeCP2 to maintain its structural integrity, whereas the substituted amino acids such as arginine fail to maintain the required structural arrangement (Figs 1B and 6D and Supplementary Table S1). The properties shared between proline and alanine may explain the relatively mild effect on the mobility dynamics for MeCP2 P152A (Fig. 4B) and on clinical severity (Fig. 5). Another example of a relatively mild phenotype at a structurally important residue is the A140V mutation, which is present within the middle of the alpha helix close to R133. Alanine, which has a non-polar, aliphatic methyl side chain, has very close properties in comparison with valine, except that valine has a slightly bulkier side chain due to a propyl group attached to C2. On another distal part of the MBD, N126I showed a similar clustering defect, likely due to the substitution of the polar and hydrophilic asparagine with a non-polar hydrophobic isoleucine (Figs 1B and 6F and Supplementary Table S1). Together, this may highlight the importance of exposed hydrophilic residues on distal parts of MBD in chromatin clustering, which are either required to maintain the structure of MeCP2 or involved in recruiting other proteins.

Evaluation of newly identified MeCP2 MBD missense variants through the interpretation of the chromatin binding and clustering effects, as reported here, may lead to improved and individualized diagnostics. Personalized therapeutic interventions may be developed based on our observations here, by selecting compounds on the basis of exclusive binding to the mutant versus wild-type MECP2 protein, then screening for improvements to the binding/chromocenter organization dynamics.

## Methods

### Experimental design

The three-dimensional structure of MeCP2 MBD shows missense variants at: 1) the proximal part of MBD, which binds directly with the methylated DNA; and 2) the distal part of MBD, which appears not to be involved directly in DNA binding (Fig. 1A).

In this study we selected twelve MeCP2 missense mutations: R106W, R111G, N126I, R133C, R133L, A140V, P152A, P152H, P152R, F157I, T158M and R167W. Out of these twelve, there are two “hotspot” residues (R133, R111) that may interact with the DNA directly, and are present on or close to the MBD-methylated DNA interface, and residues (N126, P152) that are present on the distal part of the MBD of MeCP2 (Fig. 1A). R106W, F157I and R167W were selected due to their interesting position within the 3D structure. R106W, is present in the middle of a β-sheet, F157I is adjacent to T158M (Fig. 1A), whereas R167W is present a few residues away from T158M (Supplementary Figure S1); all three maybe crucial for DNA-MeCP2 binding and/or clustering of DNA (Fig. 1A and Supplementary Figure S1). To show that the binding and clustering effect is entirely due to the missense mutations in MBD of MeCP2, we generated two constructs, with a C-terminal deletion (PP390-391del) and an N-terminal mutation (MeCP2_e1: A2V), both with the normal MBD- as controls in addition to the wild type (WT) MeCP2. MECP2 is a major organizer of chromatin structure, and heterochromatin in particular, and helps to re-organize DNA into chromocenters^32^. For all co-localization experiments, after fixation, cells were stained using DAPI which binds AT base pairs at the center of the DNA duplex^33^. AT base pairs are present across the genome, but are enriched in heterochromatin compared to euchromatin. In order to assay the effects of MECP2 mutation on binding to and clustering of chromocenter DNA, we need to identify chromocenters. For this we have used co-localization of DAPI and MECP2-GFP.

### Cell culture, vector system and mutagenesis

The mouse skeletal muscle cell line (myoblast) C2C12 (ATCC, Manassas, VA) was used as the cell system for this study due to the relatively prominent chromocenters and low endogenous MeCP2 expression^22^. In order to express MeCP2 in a mammalian system, the full length *MECP2_E1* gene was amplified using reverse transcriptase polymerase chain reaction followed by cloning into the expression construct pcDNA3.1 CT-GFP-TOPO (placing GFP at the C-terminal end of MeCP2), according to the manufacturer’s instructions (Life Technologies, Carlsbad, CA). Mutations were introduced through PCR based site-directed mutagenesis according to the manufacturer’s instructions (QuikChange Lightning Site-Directed Mutagenesis Kit, Agilent Technologies, Santa Clara, CA), in order to generate the MeCP2 missense substitutions R106W, R111G, N126I, R133L, R133C, A140V, P152A, P152R, P152H, F157I, T158M, also an N-terminal missense substitution A2V, and a C-terminal deletion P390_P391del, for use as positive controls, in addition to the wild type (WT) MeCP2.

### Fluorescence confocal microscopy

Wild type and mutant MeCP2_E1-GFP fusion protein was expressed in C2C12 cells (mouse myoblasts; ATCC, Manassas, VA) after cloning in the pcDNA3.1 CT-GFP-TOPO vector (Life Technologies). Cells were transected with a polyethylenimine transfection reagent (jetPEI, PolyPlus, Illkirch, France) following the manufacturer’s instructions. Cells were fixed using 4% paraformaldehyde followed by 2X washes of ice-cold phosphate buffer saline (PBS), and the transfected cells were then stained with the AT-selective DNA dye DAPI (4′-6′-diamidino-2-phenylindol). Fixed-cell confocal microscopy was used to study MeCP2 accumulation at chromocenters. Z-stacks were acquired with a frame size of 1024 × 1024 pixels of MeCP2-GFP expressing fixed C2C12 cells.

### Identification of single cell nuclei and chromocenters

Identification of single cell nuclei and chromocenters was performed by intensity-based thresholding and by implementation of the Water algorithm^34^. GFP fusion protein and heterochromatin were captured by confocal microscopy (Olympus FV1200) using 63×/1.3 NA oil objective at 405 nm diode pumped solid state for DAPI, and 488 nm Argon for GFP.

### Fluorescence recovery after photobleaching

To study protein binding dynamics in live cells, C2C12 cells were cultured in chambered cover glass culture plates (Nalge Nunc International, Rochester, NY). Normal and mutant MeCP2_E1-GFP proteins were transfected and expressed as described above. Fluorescence recovery after photobleaching (FRAP) was used to capture binding and diffusion dynamics. Live cell images were captured on an Olympus FV1200 confocal microscope using 63x/1.3 NA oil objective at 488 nm argon for GFP. Ten independent FRAP experiments in the temperature (37 °C)- and CO_2_ (5%) controlled incubation chambers were performed on the recombinant protein for each of the MeCP2 missense substitutions P152A, P152A, P152H, T158M, R111G and wild type MeCP2 in the C2C12 cells. FRAP imaging series of all the GFP fusion protein constructs were recorded as follow: 25 pre-bleach frames followed by 200 μs bleach time with 405 nm laser line at 100% transmission, and at least 200 post-bleach frames were recorded at equal time intervals using time lapse imaging. For confocal time-lapse imaging, a frame size of 512 × 512 pixels and 488 nm laser excitation with 0.05 transmission were used to capture GFP-tagged protein post bleach recovery frames.

### Data analysis

The cell images were captured using OlympusFluoView software. Pearson’s correlation coefficient (PCC) for the co-localization of chromocenters (DAPI) and recombinant protein (GFP) were calculated using FluoView software. The identified chromocenters’ size and their number were calculated using Fiji Particle Analysis plugin^35^. FRAP data was analyzed on an automated MATLAB based windows application, easyFRAP^37^. The descriptive statistical data analysis was calculated using Microsoft Excel and two tailed Mann Whitney non-parametric U test for statistical significance were calculated using GraphPad online tool. The questionnaire used to calculate clinical severity score (CSS) can be found in Supplementary Material. Written, informed consent was provided for all human subjects, and approval for use of human subjects was given by the respective institutions’ research ethics board (Centre for addiction and Mental Health (CAMH) and Civitan International Research Center). Where study participants were minors or not able to provide consent, consent was obtained from a parent or legal guardian.

All experiments were performed in accordance with under relevant ethics and laboratory safety guidelines, under approved experimental protocols (CAMH Research Ethics Board protocol #194/2005, and CAMH Biosafety license #2014-02).

## Additional Information

**How to cite this article**: Sheikh, T. I. *et al*. From Function to Phenotype: Impaired DNA Binding and Clustering Correlates with Clinical Severity in Males with Missense Mutations in *MECP2. Sci. Rep.* **6**, 38590; doi: 10.1038/srep38590 (2016).

**Publisher's note:** Springer Nature remains neutral with regard to jurisdictional claims in published maps and institutional affiliations.

## Supplementary Material

Supplementary Material

Supplementary Video 1

Supplementary Video 2

Supplementary Video 3

Supplementary Video 4

Supplementary Video 5

Supplementary Video 6

## Figures and Tables

**Table 1:** Clinical features of study participants.

	Case I	Case II	Case III	Case IV	Case V	Case VI	Case VII	Case VIII	Case IX	Case X^a^	Case XI
Genotype	T158M	T158M	T158M	T158M	R133C	F157I	N126I	P152H	R167W	A140V	P152A
Condition at birth	Normal	Normal	Normal	Normal	Normal	Abnormal	Abnormal	Unknown	Normal	Normal	Normal
First Reported abnormality	<10 M	4–5 months	postnatal	postnatal	~8 M	Neonatal period	Early infancy	4 YR	1.5 YR	2 YR & older	None^b^
Development	Delayed	Delayed	Delayed	Delayed	Delayed	Minimal	Minimal	Delayed	Delayed	Delayed	Normal
Head Circum-ference	Lower than normal	Lower than normal	Lower than normal	Not known	Normal at birth; <2% age 2 YR	Normal at birth; <2% age 9 M	Normal at birth; <2% age 6 M	Lower than normal	Normal	Normal	Lower than normal
Muscular abnorm-alities	Hypotonia	Hypotonia	Hypotonia	Hypertonia, limb spasticity	Hypotonia	Hypertonia	Hypotonia, then hypertonia	General muscle weakness	Normal	Hypotonia, then spastic paraparesis	None^c^
Motor skills	Never learned	Never learned	Poor head control	Regression of motor skills	Regression at age 2.5 YR	Never learned	Minimal skills	Regression	Mild delay	Mild delay	Normal
Epileptic Seizures	Yes	Yes	Not reported	Yes	Yes	Absent	Absent	No	Absent	Described in some	No
Age (Years)	Died at ~3	Died at ~1.5	Died at ~ 0.92	Died at ~1.2	13.33	11.0^d^	Died 3.17	~40	21.17	27 – middle age	~28
Reason of death	Respiratory infection	Respiratory infection	Respiratory infection	Respiratory infection	N.A	N.A	Respiratory failure	N.A	N.A	N.A	N.A
Language skills	N.A	N.A	N.A	N.A	None	None	None	Several words	Short phrases	Short phrases or simple sentences	Able to write and read
CSS^e^	36	43	31	47	37	37	36	18	12	10^f^; 1–5^g^	1
Ref.	15	15	16	17	Present study	Present study	Present study	Present study	Present study	Numerous^41^,^42^,^43^	18

^a^Here we report an amalgamation of data for several Ala140Val cases from the literature^41^,^42^,^43^ plus one (Case X)from the RDCRC Natural History Study database.

^b^No physical abnormality.

^c^No muscular abnormality reported.

^d^On home ventilator.

^e^Clinical severity scores.

^f^RDCRC Natural History Study Database.

^g^based on limited data from literature^41^,^42^,^43^.
