# Supplementary material for: From Function to Phenotype: Impaired DNA Binding and Clustering Correlates with Clinical Severity in Males with Missense Mutations in MECP2
Source: Sci Rep. 2016 Dec 8;6:38590. doi: 10.1038/srep38590 (PMC5144150; doi:10.1038/srep38590)
Supplement: Supplementary Material [file srep38590-s1.docx]

**Supplementary material for manuscript**

**Title**

**From Function to Phenotype: Impaired DNA Binding and Clustering Correlates with Clinical Severity in Males with Missense Mutations in *MECP2***

**Authors**

Taimoor I. Sheikh^1,2^, Juan Ausio^3^, Hannah Faghfoury^4^, Josh Silver^4^, Jane B. Lane^5^, James H. Eubanks^6,7,8^, Patrick MacLeod^9^, Alan K. Percy^5^, John B. Vincent^1,2,10^*

**Legend to Videos**

**Supplementary Videos 1:** Real time FRAP recovery dynamics of P152A (Video 1), P152H (Video 2), P152R (Video 3), T158M (Video 4), R111G (Video 5) and wild type MeCP2 (Video 6 ). showing a variable recovery rates and mobility dynamics.

**Supplementary Videos 1:** Real time FRAP recovery dynamics of MeCP2 p.P152A.

**Supplementary Videos 2:** Real time FRAP recovery dynamics of MeCP2 p.P152H.

**Supplementary Videos 3:** Real time FRAP recovery dynamics of MeCP2 p.P152R.

**Supplementary Videos 4:** Real time FRAP recovery dynamics of MeCP2 p.T158M.

**Supplementary Videos 5:** Real time FRAP recovery dynamics of MeCP2 p.R111G.

**Supplementary Videos 6:** Real time FRAP recovery of wild type MeCP2

**Supplementary Figures and Tables**

**Figure S1**

**
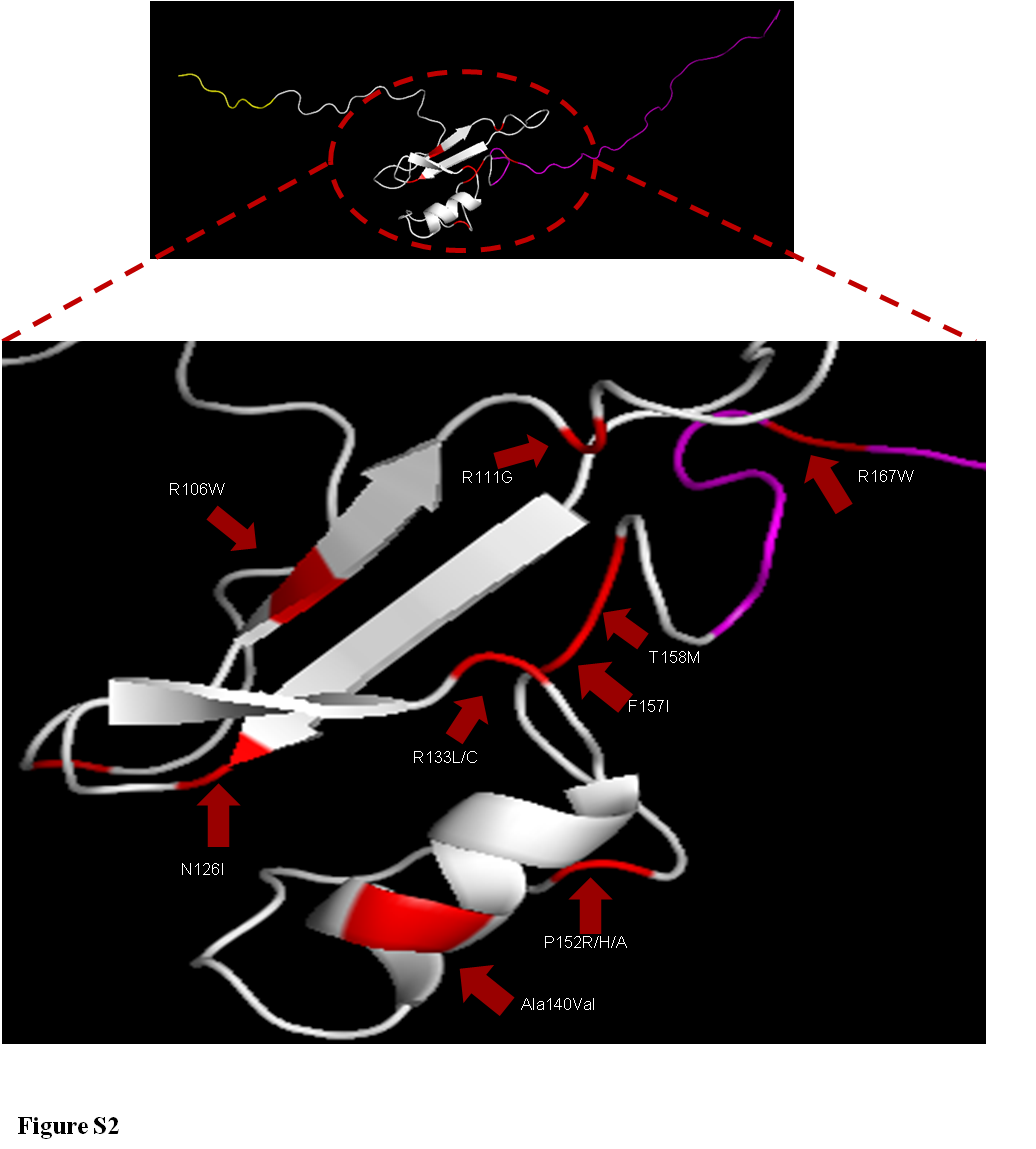
**

**Legend to Supplementary figure**

**Figure S1.** A homology model of unbound MeCP2 were generated using Phyre 2 (41) and represented in PyMol Molecular Graphic System showing NTD (yellow)-MBD (white)-ID (Magenta). Amino acid location of substituted residues are shown in red including.

**Supplementary table**

**Table S1.** Tabular illustration of the chemical properties of wild type and substituting amino acids.

| MeCP2 Mutation | Wild type amino acid properties | Substituting amino acid properties |
| --- | --- | --- |
| R133L | **Positive**  **Methylene**  **Aliphatic** | **Nonpolar**  **Methylene**  **Aliphatic** |
| T158M | **Polar**  **Hydroxyl**  **Aliphatic** | **Nonpolar**  **Sulfur**  **Aliphatic** |
| R133C | **Positive**  **Methylene**  **Aliphatic** | **Polar**  **Sulfur**  **Aliphatic** |
| R111G | **Positive**  **Methylene**  **Aliphatic** | **Nonpolar**  **Methylene**  **Aliphatic** |
| R106W | **Positive**  **Methylene**  **Aliphatic** | **Nonpolar**  **Methylene**  **Aromatic** |
| F157I | **Nonpolar**  **Methylene**  **Aromatic** | **Nonpolar**  **Methylene**  **Aliphatic** |
| P152A | **Nonpolar**  **Methylene**  **Imino** | **Nonpolar**  **Methylene**  **Aliphatic** |
| P152R | **Nonpolar**  **Methylene**  **Imino** | **Positive**  **Methylene**  **Aliphatic** |
| P152H | **Nonpolar**  **Methylene**  **Imino** | **Positive**  **Methylene**  **Aromatic** |
| N126I | **Polar**  **Amide**  **Aliphatic** | **Nonpolar**  **Methylene**  **Aliphatic** |
| A140V | **Nonpolar**  **Methylene**  **Aliphatic** | **Nonpolar**  **Methylene**  **Aliphatic** |
| R167W | **Positive**  **Methylene**  **Aliphatic** | **Nonpolar**  **Methylene**  **Aromatic** |

**Supplement Text**

Detailed clinical description of study participants.

**Case I, II, III, IV (p.Thr158Met)**

See Table 1(15-17).

**Case V (p.Arg133Cys)**

The participant was 14 years of age at the time of the report, was born at term with appropriate growth parameters. His head circumference was in the 25^th^centile at two months (the earliest measurement), but declined to just above the 2^nd^ centile by age two where it has remained. He learned to roll over, but made few gains in gross motor skills beyond this. He walked briefly with support at eighteen months. He held a bottle and reached for a toy at six months, developed a pincer grasp briefly at twenty-eight months, then losing it within a month. He finger-fed for a period of time until this ability was lost at thirty months. Expressive language developed albeit slowly, being able to babble at thirty months and losing this at sixty months. He has always fixed and followed, and quieted to voice, and briefly inhibited to ‘No’ at eleven months. Generalized epilepsy began at age two and has been virtually intractable. Drooling, bruxism, gastroesophageal reflux, and constipation have been present since age two. Repetitive rubbing of his nose began at age two and hand clasping at age four. Frequent breath holding and hyperventilation are present during wakefulness. Scoliosis was noted at age thirty months, presently with Cobb angle greater than sixty degrees. He is fed by gastrostomy tube and remains well-grown, with a BMI of 19.8. He has an *MECP2* mutation at NM_004992.1:c.397C>T (p.Arg133Cys). His mother has then same mutation but has favorable X chromosome inactivation.

**Case VI (p.Phe157Ile)**

The participant was 11 years of age at the time of the report, was born at term, but was abnormal in the nursery with frequent myoclonic movements, but no evidence of electroencephalographic abnormalities. Head circumference was at the 50^th^ centile at birth, but rapidly decelerated to the 2^nd^ centile by nine months and remained just below the 2^nd^ centile thereafter. He made limited developmental progress, being able to sit when placed at three months, and to finger feed and reach for a toy at twelve months. He lost these skills just after 13 months. He never developed a social smile or any form of expressive language, but did fix and follow and quiet to voice. He always liked being held and remained aurally responsive but stopped being visually responsive in the first few months of life. He had recurrent rubbing of his nose that began about his first birthday. He developed frequent drooling, bruxism, gastroesophageal reflux, and constipation between one and two years, but did not have periodic breathing initially. Scoliosis was evident in the first year and exceeded a Cobb angle of thirty degrees by age 8. By age three he had significant respiratory pauses requiring intubation and the parents elected to maintain him on a home ventilator thereafter. He presently sleeps much of the time (>22 hours per day), has frequent myoclonic movements, and while his EEG is slow, he had minimal epileptiform discharges. He has an*MECP2* mutation at NM_004992.1:c.469T>A (p.Phe157Ile). His mother does not have this change in peripheral blood.

**Case VII (p.Asn126Ile)**

The participant was born at term with appropriate growth parameters. He was noted to be ‘not completely normal’ during early infancy, with lethargy, marked reduction in muscle tone, poor feeding, and disrupted sleep pattern. He developed no gross motor skills such as sitting or pulling to a stand, but reached for a toy at six months with a modified pincer grasp which he retained. He did have a social smile by three months and fixed and followed. He also quieted to voice at one month, but appeared to lose response to sound shortly thereafter, and inhibited to ‘No’ at eight months, retaining this. At one month he developed periodic breathing with hyperventilation and apnea in a recurring pattern. At two months he developed hand stereotypies with repetitive nose rubbing at two months, hand washing movements at four months, and hand mouthing at five months. He developed persistent drooling, bruxism, gastroesophageal reflux, and constipation quite early as well. Scoliosis was noted by the end of his first year. Head control improved, but was never normal. A gastrostomy tube was placed at six months with marked improvement in interaction and sleeping. He did have deceleration in the rate of normal head circumference growth, being at the 50^th^ centile at one month, falling below the 2^nd^ centile at six months, and being markedly below the 2^nd^ centile at two years (43.6 cm). Genetic testing at eight months revealed a point mutation in *MECP2*at NM_004992.1:c.377A>T (p.Asn126Ile). His mother did not have the same abnormality, indicating a *de novo* mutation. A cranial MRI was unremarkable. He failed to meet consensus criteria for RTT due to the absence of normal development and the failure to regress. His condition steadily worsened over the following two years with reduced interaction, appearance of dystonia in his ankles, and marked worsening of scoliosis to >60 degrees. At just over three years of age, he developed a significant respiratory illness from which he did not recover.

**Case VIII (p.Pro152His)**

The participant is a 40 year old male at time of this report with a long history of neurological problems. Full family history is not known, but the subject’s biological mother is reported to have had intellectual impairment, and lived in an assisted living facility. He was raised in foster care, and had no medical history prior to age 4, but was known to have had deficits at the time of adoption, including a tremor, cognitive delays and gross motor delays, needing assistance with running. Currently his weight is 56.4 Kg and head circumference is 54 cm. Brain CT showed no major structural abnormalities. Abdominal ultrasound showed cholelithiasis but no other findings, and transthoracic ECHO was also normal. The subject has had 6-7 episodes of abrupt motor decline beginning in his mid 20s. After the most recent episode he exhibited a dramatic motor regression and completely lost independent walking. There was no associated fever, illness, altered mental status, vomiting, or hospitalization. He has severe progressive kyphoscoliosis and sleep apnea. He has past medical history of gastroesophageal reflux disease, and chronic drooling. He frequently speaks in two word phrases, but is also able to speak in full sentences. He spent the first few years of elementary schooling in special education classes. A previous psychological/educational assessment at the age of 20 suggested a mental age of 5-8 years. He was also diagnosed with bipolar disorder and panic disorder at that time. Investigations to date include negative tests for Huntington’s disease, Friedreich’s ataxia and Fragile X, Niemann-Pick disease type C (NPC1/NPC2b genes), and spinocerebellar degeneration. He has not had any documented seizures, cardiac symptoms or gastrointestinal problems.

Microarray studies revealed a small hemizygous 1q32 deletion (chr1:199,305,973-199,618,728; hg18) of ~0.313Mb, resulting in the loss of one copy of three Refseq genes (*TMEM9*, *IGFN1*, *LAD1*) and three OMIM morbid genes (*CACNA1S*, *PKP1*, *TNNT2*). This region does not overlap with the known 1q21.1 deletion syndrome, or other known chromosomal abnormalities, and does not overlap with deletions in the Database of Genomic Variants (DGV: dgv.tcag.ca), although several smaller deletions do map to this region. *CACNA1S* and *TNNT2* genes have been associated with dominant hypokalemic periodic paralysis and dominant cardiomyopathy respectively, but clinically he does not have any of these signs. The subject has a mutation in the *MECP2* gene at NM_04992.1:c.455C>A (p.Pro152His) which has never been reported to date. In summary he is a 40 year old male with partial loss of acquired purposeful hand movements (tremor), some loss of acquired spoken skills, dyspraxia, sleep disorder, scoliosis and with intellectual disability and late-onset regression.

**Case IX (p.Arg167Trp)**

The participant was 22 years of age at time of report, was born at term with appropriate growth parameters. Hs head circumference at birth was at the 50^th^ centile where it has remained. He was somewhat slow in gross motor development not learning to walk independently until eighteen months and not being able to navigate steps down until three years. Fine motor development was also delayed, not being able to finger feed until twenty-four months. Single words appeared at thirteen months, disappeared at fourteen months, and reappeared at five years. He spoke in phrases first at age 10. Receptive language was also delayed considerably. For example, he did not follow a command without a gesture until age eight. Seizures were noted at age four, but have not been present for many years while on antiepileptic agents. He has never had periodic breathing but developed a number of hand stereotypies at age three including hand clapping/tapping, hand mouthing, and finger rubbing movements. These are limited to finger rubbing movements when he is not otherwise occupied at present. Scoliosis was noted at age two. An *MECP2* mutation was reported at age 12 at NM_04992.1:c.499C>T (p.Arg167Trp). Presently, he is in good health overall, and requires medication for mood stabilization, but is otherwise doing well in a group home.

**Case X (p.Ala140Val)**

The participant is a 31 year-old male with a mutation in *MECP2* at NM_04992.1:c.419C>T, p.Ala140Val. He does not meet criteria for Rett syndrome in that he did not lose hand use or communication, develop stereotypic movements, or have deceleration in head growth. A diagnosis was made at age 20 when the mutation in *MECP2* was detected. His early history is unclear as he was adopted at age 5. Gross motor skills were retained except for walking, however independent walking stopped at age 20. Fine motor skills were also retained except he stopped using a pincer grasp at age 20. He was limited to single word use at an unknown age, yet continues to be able to use single words and familiar phrases. Receptive language was retained overall. He has never had seizures, irregular breathing patterns, or bruxism, but has had persistent difficulties with constipation since age 15 years and gastroesophageal reflux since age 20. He did have hand washing movements beginning at age 5 years. Scoliosis is described but its date of origin is unknown. On examination, he gave persistent eye contact and verbalized with meaning, said multiple words with meaning, and screamed intermittently, but was not self-abusive. He had a normal breathing pattern and no hand stereotypies. He was right handed and used hand grasping to take objects. He sat independently but required assistance while walking. He did have moderate scoliosis with truncal rotation to the right. Muscle tone was increased, but strength, bulk, and muscle stretch reflexes were normal. He was noted to have dystonia, truncal

titubation, and a mild tremor. His skin temperature was normal. He has been remarkably stable over the past 10 years. Also see References 32-34.

**Case XI (p.Pro152Ala)**

See Table 1(18).
